# Multicomponent synthesis of some new (1*S*,4*S*)-2,5-diazabicyclo[2.2.1]heptane-dithiocarbamates and their *in vitro* anti-proliferative activity against CaSki, MDA-MB-231 and SK-Lu-1 tumour cells as apoptosis inducing agents without necrosis

**DOI:** 10.1080/14756366.2017.1363197

**Published:** 2017-09-03

**Authors:** Sujay Laskar, Luis Sánchez-Sánchez, Manuel López-Ortiz, Hugo López-Muñoz, María L. Escobar-Sánchez, Arturo T. Sánchez, Ignacio Regla

**Affiliations:** a Lab. de Síntesis de Fármacos, Laboratorio 9 UMIEZ, Facultad de Estudios Superiores Zaragoza, Universidad Nacional Autónoma de México, Ciudad de México, Mexico;; b Lab. Biología Molecular del Cáncer, Laboratorio 2 PB UMIEZ, Facultad de Estudios Superiores Zaragoza, Universidad Nacional Autónoma de México, Ciudad de México, Mexico;; c Depto. Biología Celular, Lab. Microscopía Electrónica, Facultad de Ciencias, Universidad Nacional Autónoma de México, Ciudad de México, Mexico

**Keywords:** Multicomponent, (1*S*,4*S*)-2,5-diazabicyclo[2.2.1]heptane, dithiocarbamate, antiproliferative, apoptosis, necrosis

## Abstract

Identification of a new class of antitumor agent capable to induce apoptosis without triggering necrotic cell death event is challenging. The present communication describes the multicomponent synthesis of seven new (1*S*,4*S*)-2,5-diazabicyclo[2.2.1]heptane-dithiocarbamates and their *in vitro* antiproliferative activity on cervical cancer cell line (CaSki), breast cancer cell line (MDA-MB231), lung cancer cell line (SK-Lu-1) and human lymphocytes. Among the synthesized dithiocarbamates, compound **9e** displayed significant antiproliferative activity without inducing any necrotic cell death (both on tumour cells and lymphocytes) and induced apoptosis in tumor cells by the caspase dependent apoptotic pathway. The compound **9e** also exhibited greater tumor selectivity than human lymphocytes. *In silico* ADME predictions revealed that compound **9e** has the potential to be developed as a drug candidate. Rapid chemical modifications of this lead are thus highly necessary for further investigation as a drug like safer antitumor candidate and also to achieve compounds with better activity profile.

## Introduction

During the design of safer antiproliferative agents, it would be desirable to take into account the actual side effects related with different cell death processes^1^. Apoptosis and necrosis represent two fundamental types of cell death processes^2^. Apoptotic cell death is a regulated cellular mechanism, however the plasma membrane retains the integrity during the process^3^. In contrast, necrotic cells undergo plasma membrane rupture, nuclear and cellular swelling^4^. Necrosis is usually followed by an inflammatory response to the released cellular contents, often resulting in further tissue damage^5^. Majorly of the cytotoxic drugs not only target neoplastic cells but are also toxic to normal cells and organs. As a result, chemotherapy is always associated with adverse effects, including substantial impacts on the immune system. Such kind of undesired effects very often are detrimental to the health of the patients.

**Figure 1.**
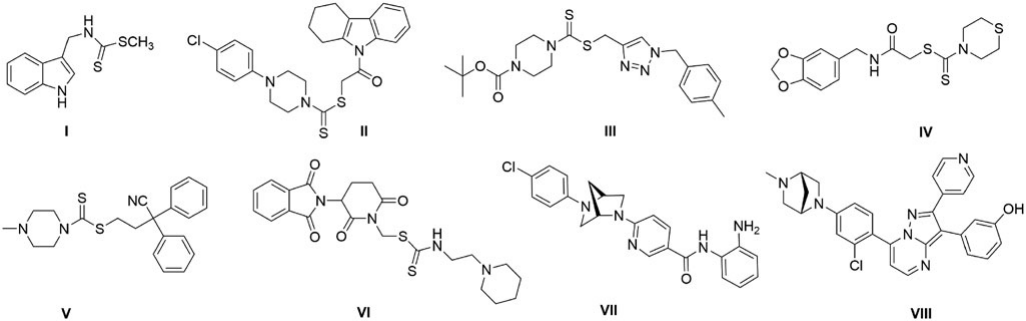
Structures of some dithiocarbamates and (1*S*,4*S*)-2,5-diazabicyclo[2.2.1]heptanes displaying potent anticancer activity.

Literature revealed that many dithiocarbamate derivatives displayed potent anticancer activity (Figure 1) both in *in vitro* and *in vivo* model and might act by induction of apoptosis^6^. Molecular hybridization technique had been widely adopted in designing new dithiocarbamate cytotoxic agents which includes tetrahydrocarbazole^7^, 1,2,3-triazoles^8^, quinazolines^9^, emetine^10^, chromones^11^, benzodioxole^12^ dithiocarbamate derivatives, etc^13^ ^,^ ^14^. However, the molecular hybridization of dithiocarbamate with bridged bicyclic compounds to achieve new class of antiproliferative agents have not been reported so far. Among the bridged *N*-heterocycles conformationally restricted rigid piperazine homolog 2,5-diazabicyclo[2.2.1]heptane has been extensively used in medicinal chemistry for synthesizing potent drug candidates^15^. Surprisingly, only two literatures (from Merck Research Laboratories and Wyeth Research) are currently available applying 2,5-diazabicyclo[2.2.1]heptane to achieve antitumor agents (Figure 1, VII, VIII)^16^ ^,^ ^17^. In the context of current drug discovery strategies, empirical approaches without particular target largely depends on the quality of the newly synthesized molecules in respect to molecular complexity and diversity which could be built easily through multi-component reactions^18^. Until now, there are no reports available for the functionalization of 2,5-diazabicyclo[2.2.1]heptanes through multicomponent reaction pathway to achieve rapid diversity in the framework.

In view of the above, here in we have reported the first multicomponent synthesis of seven new (1*S*,4*S*)-2,5-diazabicyclo[2.2.1]heptane-dithiocarbamates and *in vitro* biological evaluation of their antitumor activity, apoptosis-inducing effect, necrotic effect, selectivity and ADME profiling.

## Experimental

### Materials and methods

Melting points were determined in open capillaries in a MEL-TEMP^®^ melting point apparatus. ^1^H and ^13^C NMR spectra were obtained at 300/400 MHz and 75/100 MHz respectively using Mercury-Plus400 spectrometer and CDCl_3_/DMSO-d_6_ as solvent; chemical shifts were recorded in parts per million (ppm) with TMS as the internal reference. Mass spectra (FAB-MS) were measured on a MS Station, MARCA JEOL, JMS-700 equipment and signals were given in *m/z*. Optical rotations were determined in a Perkin-Elmer 341 polarimeter using a 1-dm cell path length (sodium D-line 589 nm), at 20 °C sample compartment temperature.

### Chemistry

#### Synthesis of (1S,4S)-2-Boc-2,5-diazabicyclo[2.2.1]heptane (7, Scheme 2)

Free base **5** of the (1*S*,4*S*)-2-benzyl-2,5-diazabicyclo[2.2.1]heptane dihydrobromide salt **4** ^19^ was obtained by the treatment of sodium methoxide (in methanol) solution and was used in the next stage without storage. In a 250 ml round bottom flask, 28 g (148.72 mmol) of (1*S*,4*S*)-2-benzyl-2,5-diazabicyclo[2.2.1]heptane in 200 ml of dichloromethane was added and flask was placed in an ice-bath. About 41.24 g (188.52 mmol) of di-*tert*-butyl dicarbonate was added in portions into the reaction flask followed by the addition of 26.25 ml (188.52 mmol) triethylamine. The reaction flask was slowly warmed to room temperature and the stirring was continued. After completion of the reaction (as monitored by TLC), the reaction mixture was washed with distilled water (3 × 200 ml), dried over sodium sulphate and concentrated under vacuum to get white solid product **6** with 90% yield (53 g). After that, 53 g (183 mmol) of **6** was dissolved in 300 ml dry methanol and placed in a 500 ml hydrogenation flask (60 psi) in presence of 10% by weight of Pearlman’s catalyst. After completion of the hydrazinolysis, the catalyst was filtered off and the filtrate containing the product was concentrated to obtain a white solid as *N*-Boc-DBH **7** with 90% yield (40 g).

#### Multicomponent synthesis of (1S,4S)-2,5-diazabicyclo[2.2.1]heptane-dithiocarbamates (9a–9g, Scheme 3)

An oven-dried screw cap reaction tube was charged with a magnetic stir bar, *N*-Boc-DBH (1 mmol), catalyst (MgO; 0.5 mmol) and 3 ml methanol. Carbon disulphide (1.5 mmol) was added drop wise to the stirred mixture at 0 °C temperature. After 30 min of stirring, reactant **8** (**8a**–**8g**, 1 mmol) was added slowly to the stirred reaction mixture, and the stirring was continued for overnight at ambient temperature. The progress of the reaction was monitored by thin-layer chromatography (TLC). On completion of the reaction, methanol was evaporated on a rotary evaporator and the product was extracted with dichloromethane followed by column chromatographic purification over silica gel (heptane/ethyl acetate) to provide the pure product. Although this procedure was described on the mmol scale, gram-scale reactions also provided uniform results.

### Biological activity

#### Cell proliferation assays

CaSki (cervical cancer cell line), MDA-MB-231 (breast cancer cell line) and SK-Lu-1 (lung cancer cell line) were purchased from the American Type Culture Collection (ATCC Rockville, MD) and assays were performed by seeding 7500 cells/well in 96-well tissue culture plates at a volume of 100 µL of RPMI-1640 medium supplemented with 5% NCS per well. Cells were allowed to grow for 24 h in the culture medium prior to exposure with the compounds. Also, 1% of vehicle (ETOH or ETOH:DMSO 1:1) was added to the control cells. Antiproliferative activity was determined after 24 h by crystal violet staining^21^. Cell counts were determined by measuring absorbance at 590 nm in an enzyme-linked immunosorbent assay (ELISA) plate reader.

#### Determination of necrotic effect

Lactate dehydrogenase (LDH) release from cells was determined using LDH assay kit to confirm cell necrosis^22^. The experiments were carried out following the manufacturer’s protocol (CytoTox 96^®^ Non-Radioactive; Cytotoxicity Assay; Promega, Littleton, CO).

#### CFSE-labelling assay

Lymphocytes were obtained from peripheral blood of healthy human volunteers, and isolated by density gradient centrifugation and cultured in 96-well plates. Lymphocyte proliferation was induced with phytohemaglutinin and were treated with the compounds. The proliferation was evaluated after 72 h by the incorporated CFSE-labelling assay^23^.

#### Immunolocalisation of active caspase-3 by fluorescence microscopy

Cells were cultured in glass coverslips and treated with the compounds during 24 h. The cells were fixed in 2% paraformaldehyde. The cells were permeabilised with 0.05% Triton X-100 and incubated with anti-active caspase-3 antibody (Novous Biologicals, Littleton, CO). Next, the samples were washed and incubated with a secondary goat anti-rabbit antibody with fluorescein isothiocyanate. Finally, they were counterstained with 4,6-diamidino-2-phenylindole (DAPI). Immunoassays were evaluated under a Nikon Eclipse E600 Microscope and images were recorded with a Nikon Digital DXM1 200F Camera.

## Results and discussion

**Scheme 1.**
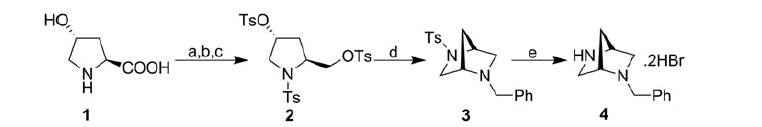
Synthesis of (1*S*,4*S*)-2-benzyl-2,5-diazabicyclo[2.2.1]heptane dihydrobromide. Reagents and conditions. (a) TsCl, Na_2_CO_3_, H_2_O, 94%; (b) NaBH_4_, BF_3_–Et_2_O, THF, 85%; (c) TsCl, C_5_H_5_N, toluene, 20 h, 83%; (d) PhCH_2_NH_2_, toluene, reflux, 96%; (e) HBr 40%, 96%.

**Scheme 2.**
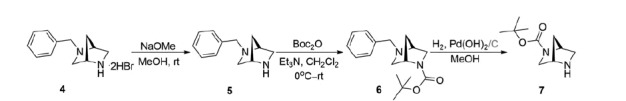
Synthesis of (1*S*,4*S*)-*tert*-butyl 2,5-diazabicyclo[2.2.1]heptane-2-carboxylate.

**Scheme 3.**
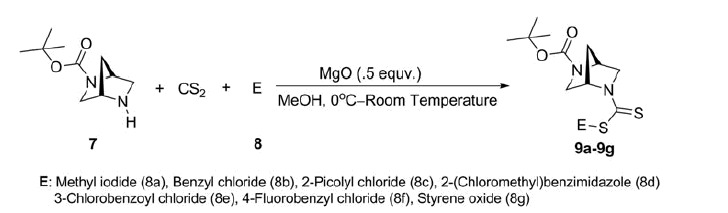
One-pot synthesis of (1*S*,4*S*)-*N*-Boc-2,5-diazabicyclo[2.2.1]heptane-dithiocarbamates.

**Figure 2.**
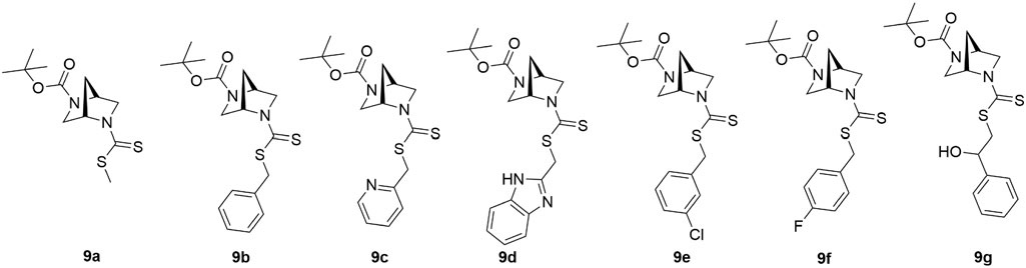
Structures of the synthesized (1*S*,4*S*)-*N*-Boc-2,5-diazabicyclo[2.2.1]heptane-dithiocarbamates.

The synthetic routes for the preparation of (1*S*,4*S*)-*N*-Boc-2,5-diazabicyclo[2.2.1]heptane-dithiocarbamates **9a–9g** have been outlined in Schemes 1–3. The starting compound **4** was synthesized according to the procedure described by Regla (our group member) and Juaristi et al.^19^ (Scheme 1). Compound **5** was treated with di-*tert*-butyl dicarbonate followed by hydrogenolysis to remove the benzyl group and afford compound **7** in good yield (Scheme 2). Multicomponent reaction strategy was then applied for straightforward synthesis of the title dithiocarbamate derivatives (Figure 2, **9a**–**9g)** in good yields following the reaction between *N*-Boc-DBH **7**, carbon disulphide and various electrophiles (**8a–8g)** in the presence of magnesium oxide as heterogeneous catalyst and methanol as solvent (Scheme 3). The reaction profile was very clean and energy efficient.

**Figure 3.**
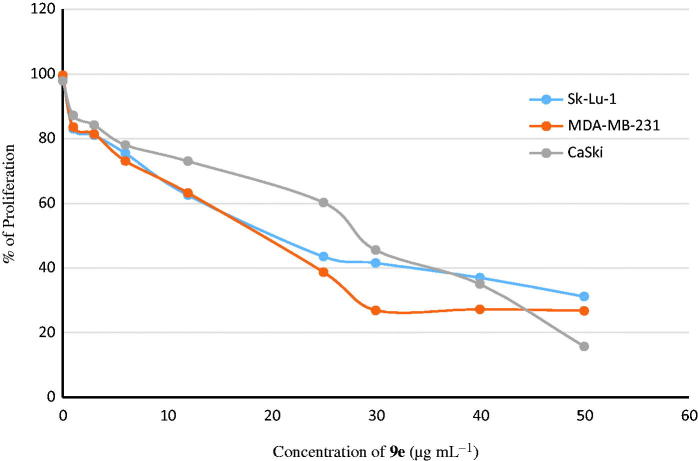
Antiproliferative dose–response curve of compound **9e** on three cancer cell.

**Figure 4.**
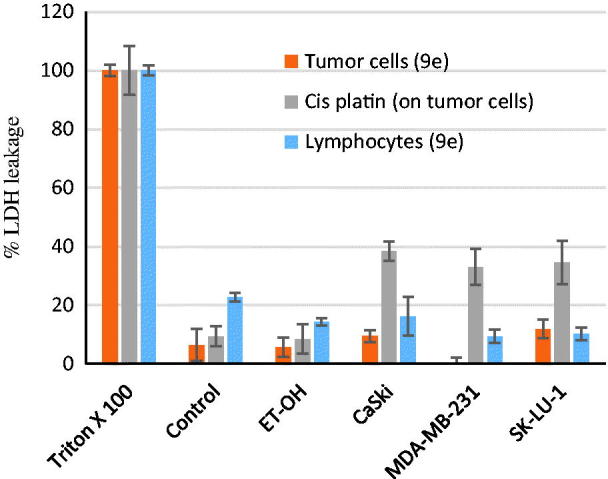
Necrotic effect of **9e** (at the IC_50_ values) on both the tumour and lymphocytes cell lines by LDH leakage assay.

**Figure 5.**
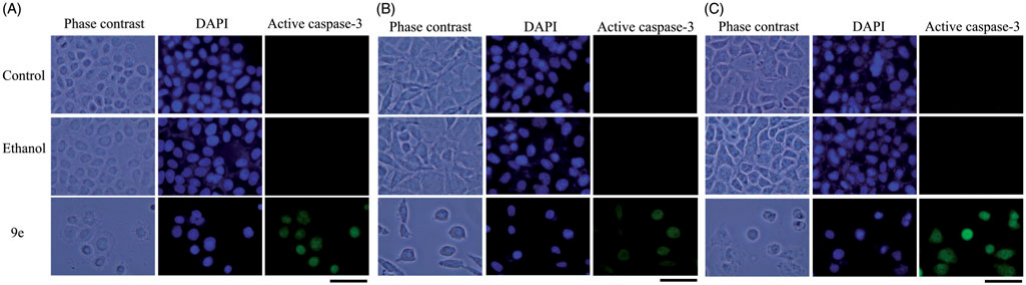
Compound **9e** induced apoptotic death. Immunodetection of active caspase-3 by compound **9e** on CaSki (A), MDA-MB-231 (B) and SK-Lu-1cultures (C).

**Figure 6.**
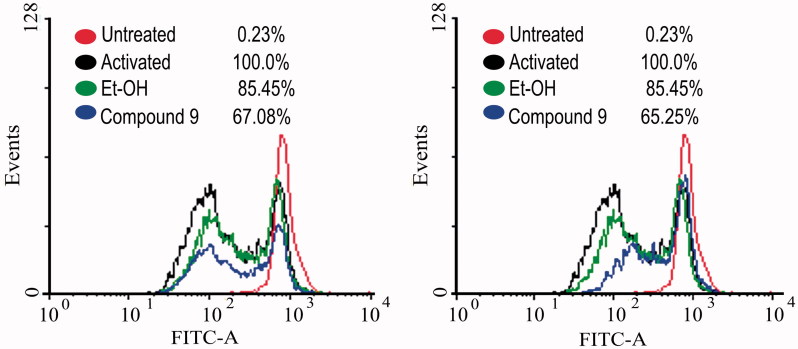
Effect of the compound **9e** on lymphocyte proliferation by CFSE-labelling assay [at the concentrations of 18 (left) and 20 (right) µg mL^−1^].

Having synthesized a variety of (1*S*,4*S*)-*N*-Boc-2,5-diazabicyclo[2.2.1]heptane-dithiocarbamates, we set out to evaluate the compounds for their possible antitumor activities. All the compounds were subjected to measure antiproliferative activity against cervical cancer cell line (CaSki), breast cancer cell line (MDA-MB-231) and lung cancer cell line (SK-Lu-1), respectively, employing ETOH and ETOH:DMSO 1:1 as a vehicle and the corresponding IC_50_ values have been shown in Table 3. Among the seven test compounds, compound **9e** showed moderate antiproliferative activity with IC_50_ values 28, 18 and 20 µg/mL against CaSki, MDA-MB231 and SK-Lu-1 cell line, respectively (Figure 3). To identify the preliminary cell death processes induced by this compound, the necrotic effect of the compound was evaluated on CaSki, MDA-MB231 and SK-Lu-1 cell lines as well as on human lymphocytes using lactate dehydrogenase (LDH) assay (Figure 4). It is our delight to mention that compound **9e** did not induce any necrotic cell death on the three tumour cells and human lymphocytes, unlike cisplatin which induced necrotic cell death (Figure 4). In the preliminary apoptosis experiment CaSki, MDA-MB-231 and SK-Lu-1 cultures were stimulated at the level of their determined IC_50_ values and the morphological changes, chromatin condensation including the formation of apoptotic bodies were determined through staining with fluorochrome 4′,6-diamidino-2-phenylindole (DAPI). Compact nuclei and apoptotic bodies were clearly observed in the cultures (Figure 5). The condensed chromatin in treated cells suggested that compound **9e** induced cell death by apoptosis in the concerned cancer cell lines. In the present study, we had detected active caspase-3 by immunodetection. Figure 5 shows that compound **9e** induced the expression of active caspase-3 in CaSki, MDA-MB-231 and SK-Lu-1 cultures, implying that apoptosis could be triggered through a caspase dependent process. It is well known that during chemotherapy the immune system is usually affected. Thus to evaluate the selectivity, the proliferation of enriched lymphocyte population (ELP) was evaluated with compound **9e** using CFSE-labelling assay (Figure 6). The results indicated that with compound **9e**, proliferative potential of lymphoid cells was not negatively affected after 72 h, implying a greater degree of antiproliferative selectivity towards malignant cell lines than with lymphocytes. *In silico* ADME profiling study revealed that compound **9e** has the potential to be developed as oral drug candidate (Table 4).

**Table 1.** Experimental data of the compounds **9a–9g.**

SL. no	Product	Time (h)	Isolated yield (%)	Melting point	Optical rotation, [α]_D_^20^
1	**9a**	10	70	109–110 °C	−193.9 (*c* 1, CH_2_Cl_2_)
2	**9b**	10	68	Viscous liquid	−150.8 (*c* 1.025, CH_2_Cl_2_)
3	**9c**	11	60	Viscous liquid	−166.84 (*c* 1.04, CH_2_Cl_2_)
4	**9d**	12	55	228–230 °C	−79.61 (*c* 0.336, DMSO)
5	**9e**	10	63	Viscous liquid	−140.4 (*c* 1.04, CH_2_Cl_2_)
6	**9f**	10	75	98–103 °C	−178.3 (*c* 178.3, CH_2_Cl_2_)
7	**9g**	10	71	Viscous liquid	−122.7 (*c* 1.046, CH_2_Cl_2_)

**Table 2.** Spectral data of the compounds **9a–9g.**

**7**	^1^H-NMR (300 MHz, CDCl_3_, ppm): *δ* = 4.33 (0.5H, *s*, –CH), 4.21 (0.5H, *s*, –CH), 3.60 (1H, *s*, –CH), 3.28–3.22 (1H, *m*, –CH_2_), 3.11-3.05 (1H, *m*, –CH_2_), 2.97–2.87 (2H, *m*, –CH_2_), 2.14 (1H, *s*, –NH), 1.62-1.56 (2H, *m*, –CH_2_), 1.35 (9H, *brs*, –CH_3_); ^13^C-NMR (75 MHz, CDCl_3_, ppm): *δ* = 154.43, 154.16, 79.39, 79.29, 57.16, 56.51, 56.11, 55.98, 52.57, 52.44, 37.03, 36.80, 28.51^20^
**9a**	^1^H-NMR (400 MHz, DMSO-d_6_, ppm): *δ* = 5.42 (0.5H, *s*, –CH), 4.99 (0.5H, *s*, –CH), 4.55–4.29 (1H, *m*, –CH), 3.74–3.63 (1H, *m*, –CH_2_), 3.49–3.40 (1H, *m*, –CH_2_), 3.28–3.13 (2H, *m*, –CH_2_), 2.58–2.56 (3H, *m*, –CH_3_), 2.02–1.75 (2H, *m*, –CH_2_), 1.40–1.38 (9H, *m*, –CH_3_); ^13^C-NMR (100 MHz, DMSO-d_6_, ppm): *δ* = 193.36 (C = S), 153.44 (C = O), 64.30, 63.74, 62.52, 62.16, 61.96, 61.40, 58.58, 58.26, 57.33, 57.16, 56.62, 56.36, 56.21, 56.00, 55.70, 54.99, 54.04, 53.63, 53.31, 52.37, 52.10, 51.76, 37.48, 36.94, 36.25, 35.74, 28.11, 28.06, 18.69, 18.56; MS (FAB+): *m/z* calculated for C_12_H_20_N_2_O_2_S_2_ [M + H]^+^: 289, found: 289
**9b**	^1^H-NMR (400 MHz, CDCl_3_, ppm): *δ* = 7.31–7.16 (5H, *m*, –ArH), 5.59 (0.5H, *s*, –CH), 4.89 (0.5H, *s*, –CH), 4.53–4.43 (3H, *m*, –CH, –CH_2_), 3.89–3.31 (4H, *m*, –CH_2_), 1.90–1.84 (2H, *m*, –CH_2_), 1.39–1.37 (9H, *m*, –CH_3_); ^13^C-NMR (100 MHz, CDCl_3_, ppm): *δ* = 193.78 (C = S), 192.78 (C = S), 154.06 (C = O), 136.14, 129.35, 128.69, 127.62, 80.32, 64.54, 63.89, 62.38, 62.28, 62.18, 61.59, 58.35, 57.55, 56.61, 56.21, 55.23, 52.34, 51.98, 41.25, 41.12, 38.02, 37.55, 36.91, 36.49, 28.50; MS (FAB+): *m/z* calculated for C_18_H_24_N_2_O_2_S_2_ [M + H]^+^: 365, found: 365
**9c**	^1^H-NMR (400 MHz, CDCl_3_, ppm): *δ* = 8.51 (1H, *d*, *J* = 4 Hz, –ArH), 7.60 (1H, *td*, *J* = 7.6 Hz, 2 Hz, –ArH), 7.45 (1H, *m*, –ArH), 7.15–7.12 (1H, *m*, –ArH), 5.62 (0.5H, *s*, –CH), 5.01 (0.5H, *s*, –CH), 4.80–4.47 (3H, *m*, –CH, –CH_2_), 3.89–3.37 (4H, *m*, –CH_2_), 1.95–1.89 (2H, *m*, –CH_2_), 1.42–1.40 (9H, *m*, –CH_3_); ^13^C-NMR (100 MHz, CDCl_3_, ppm): *δ* = 193.56 (C = S), 156.88, 154.11 (C = O), 149.65, 136.83, 123.97, 122.47, 80.44, 64.84, 64.23, 62.62, 62.56, 62.35, 61.78, 58.50, 57.64, 56.70, 56.29, 55.31, 52.41, 52.09, 52.01, 42.63, 38.11, 37.67, 37.01, 36.59, 28.58; MS (FAB+): *m/z* calculated for C_17_H_23_N_3_O_2_S_2_ [M + H]^+^: 366, found: 366
**9d**	^1^H-NMR (400 MHz, CDCl_3_, ppm): *δ* = 7.52 (2H, *brs*, –ArH), 7.21–7.19 (2H, *m*, –ArH), 5.62 (0.5H, *s*, –CH), 4.95 (0.5H, *s*, –CH), 4.91–4.50 (3H, *m*, –CH, –CH_2_), 3.95–3.31 (4H, *m*, –CH_2_), 1.95–1.85 (2H, *m*, –CH_2_), 1.44–1.40 (9H, *m*, –CH_3_); ^13^C-NMR (100 MHz, CDCl_3_, ppm): *δ* = 193.61 (C = S), 154.00 (C = O), 151.47, 122.89, 115.56, 80.66, 65.60, 65.00, 63.18, 62.97, 62.37, 58.95, 57.56, 56.67, 56.21, 55.23, 52.32, 51.93, 38.13, 37.69, 37.07, 36.63, 33.30, 33.14, 28.56; MS (FAB+): *m/z* calculated for C_19_H_24_N_4_O_2_S_2_ [M + H]^+^: 405, found: 405
**9e**	^1^H-NMR (400 MHz, CDCl_3_, ppm): *δ* = 7.31 (1H, *s*, –ArH), 7.22-7.15 (3H, *m*, –ArH), 5.59 (0.5H, *s*, –CH), 4.90 (0.5H, *s*, –CH), 4.57–4.40 (3H, *m*, –CH, –CH_2_), 3.89–3.72 (1H, *m*, –CH_2_), 3.63–3.33 (3H, *m*, –CH_2_), 1.92–1.85 (2H, *m*, –CH_2_), 1.40–1.38 (9H, *m*, –CH_3_); ^13^C-NMR (100 MHz, CDCl_3_, ppm): *δ* = 193.26 (C = S), 192.19 (C = S), 154.13 (C = O), 138.65, 138.60, 134.43, 129.95, 129.41, 127.84, 127.82, 127.58, 80.46, 64.81, 64.16, 62.60, 62.51, 62.32, 61.73, 58.45, 56.66, 56.26, 55.29, 52.41, 52.05, 40.43, 40.29, 38.11, 37.65, 36.98, 36.57, 28.58; MS (FAB+) *m/z* (%) = 399 [M+], 343, 341, 273, 241, 140, 185, 141, 125, 57; MS (FAB+): *m/z* calculated for C_18_H_23_ClN_2_O_2_S_2_ [M + H]^+^: 399, found: 399
**9f**	^1^H-NMR (400 MHz, DMSO-d_6_, ppm): *δ* = 7.45–7.41 (2H, *m*, –ArH), 7.17–7.10 (2H, *m*, –ArH), 5.43 (0.5H, *s*, –CH), 5.00 (0.5H, *s*, –CH), 4.60–4.29 (3H, *m*, –CH, –CH_2_), 3.78–3.65 (1H, *m*, –CH_2_), 3.48–3.37 (1H, *m*, –CH_2_), 3.34–3.12 (2H, *m*, –CH_2_), 2.00–1.75 (2H, *m*, –CH_2_), 1.40–1.38 (9H, *m*, –CH_3_); ^13^C-NMR (100 MHz, DMSO-d_6_, ppm): *δ* = 191.66 (C = S), 162.59, 160.17, 153.36 (C = O), 132.82, 131.09, 131.01, 115.31, 115.10, 79.25, 79.13, 64.45, 63.88, 62.60, 62.25, 62.12, 61.54, 58.58, 58.31, 57.27, 56.31, 55.92, 54.92, 52.38, 52.05, 51.66, 38.86, 37.42, 36.90, 36.16, 35.65, 28.07, 28.01; MS (FAB+): *m/z* calculated for C_18_H_23_FN_2_O_2_S_2_ [M + H]^+^: 383, found: 383
**9g**	^1^H-NMR (400 MHz, CDCl_3_, ppm): *δ* = 7.45–7.24 (5H, *m*, –ArH), 5.65–5.61 (0.3H, *m*), 5.39 (0.24H, *d*, *J* = 8 Hz), 5.02–4.97 (0.8H, *m*), 4.70–4.35 (1H, *m*), 4.15–3.27 (7H, *m*), 1.98–1.73 (2H, *m*, –CH_2_), 1.43–1.38 (9H, *m*, –CH_3_); ^13^C-NMR (100 MHz, CDCl_3_, ppm): *δ* = 193.18 (C = S), 154.10 (C = O), 143.03, 129.06, 128.97, 128.74, 128.66, 128.27, 128.15, 128.01, 127.98, 126.00, 125.97, 80.54, 73.48, 66.52, 66.27, 65.99, 65.04, 64.96, 64.84, 64.39, 62.70, 62.54, 61.95, 58.78, 58.67, 58.55, 57.60, 57.12, 56.97, 56.66, 56.30, 55.33, 55.26, 54.17, 53.74, 53.58, 52.35, 52.09, 51.99, 45.12, 44.93, 44.77, 44.63, 38.09, 37.64, 37.02, 36.62, 28.60; MS (FAB+): *m/z* calculated for C_19_H_26_N_2_O_3_S_2_ [M + H]^+^: 395, found: 395

**Table 3.** Antiproliferative activities of the synthesized (1*S*,4*S*)-*N*-Boc-2,5-diazabicyclo[2.2.1]heptane-dithiocarbamates (**9a**–**9g**).

Compound	IC_50_ (µg mL^−1^)		
	CaSki	MDA-MB231	SK-Lu-1
**9a**	346	150	300
**9b**	294	69	57
**9c**	305	100	120
**9d**	214	100	87
**9e**	**28**	**18**	**20**
**9f**	348	227	237
**9g**	137	50	40
**Cisplatin**^a^	1.67	2.37	1.36

All the experimental results are the average of three independent experiments.

a The assay was performed with a commercially available sample of Cisplatin, purchased from Sigma-Aldrich.

**Table 4.** *In silico* prediction of physicochemical pharmacokinetic properties^24^.

Code	miLogP^a^	nON^b^	nOHNH^c^	n-Violations^d^	Nrotb^e^	MW^f^
Rule	≤5	<10	<5	≤1	–	<500
**9e**	4.55	4	0	0	6	342.47

a miLogP: logarithm of partition coefficient of compound between *n*-octanol and water.

b n-ON acceptors: number of hydrogen bond acceptors.

c n-OHNH donors: number of hydrogen bonds donors.

d n-violations: number of violations according to Lipinski’s rule.

e Nrotb: number of rotatable bonds.

f MW: molecular weight.

## Conclusions

The objective of the present study was to synthesize new (1*S*,4*S*)-2,5-diazabicyclo[2.2.1]heptanes bearing dithiocarbamate moiety through MCR pathway and to study the effect on antitumor activity, apoptosis induction, necrosis as well as selectivity. One compound displayed significant antiproliferative activity against CaSki, MDA-MB-231 and SK-Lu-1 tumour cell lines (with IC_50_ values 28, 18 and 20 µg/mL, respectively) and induced apoptotic cell death through caspase-3 activation without triggering any necrosis. It also showed greater degree of tumour selectivity compared with peripheral blood lymphocytes. Thus, chemical modifications of this compound are highly necessary to afford drug like potency. Therefore, such compound could serve as promising safer antitumor agent and certainly augur well for deeper assays on mechanistic effects in the next stage of research.
